# Host Innate Immune Response of Geese Infected with Clade 2.3.4.4 H5N6 Highly Pathogenic Avian Influenza Viruses

**DOI:** 10.3390/microorganisms8020224

**Published:** 2020-02-07

**Authors:** Siyu Wu, Jianni Huang, Qiwen Huang, Junsheng Zhang, Jing Liu, Qian Xue, Weiqiang Li, Ming Liao, Peirong Jiao

**Affiliations:** 1 College of Veterinary Medicine, South China Agricultural University, Guangzhou 510642, China; 2016102809@stu.scau.edu.cn (S.W.); huangjianni1992@outlook.com (J.H.); zjs19950314@foxmail.com (J.Z.); liujing918@foxmail.com (J.L.); xq1605942919@foxmail.com (Q.X.); wqli0815@gmail.com (W.L.); 2 College of Animal Science and Technology, Guangdong Polytechnic of Science and Trade, Guangzhou 510640, China; huangqiwen0511@outlook.com

**Keywords:** H5N6, genetics, pathogenicity, host innate immune response, goose

## Abstract

Since 2014, highly pathogenic avian influenza (HPAI) H5N6 viruses have circulated in waterfowls and caused human infections in China, posing significant threats to the poultry industry and the public health. However, the genetics, pathogenicity and innate immune response of H5N6 HPAIVs in geese remain largely unknown. In this study, we analyzed the genetic characteristic of the two H5N6 viruses (GS38 and DK09) isolated from apparently healthy domestic goose and duck in live poultry markets (LPMs) of Southern China in 2016. Phylogenetic analysis showed that the HA genes of the two H5N6 viruses belonged to clade 2.3.4.4 and were clustered into the MIX-like group. The MIX-like group viruses have circulated in regions such as China, Japan, Korea, and Vietnam. The NA genes of the two H5N6 viruses were classified into the Eurasian sublineage. The internal genes including PB2, PB1, PA, NP, M, and NS of the two H5N6 viruses derived from the MIX-like. Therefore, our results suggested that the two H5N6 viruses were reassortants of the H5N1 and H6N6 viruses and likely derived from the same ancestor. Additionally, we evaluated the pathogenicity and transmission of the two H5N6 viruses in domestic geese. Results showed that both the two viruses caused serious clinical symptoms in all inoculated geese and led to high mortality in these birds. Both the two viruses were transmitted efficiently to contact geese and caused lethal infection in these birds. Furthermore, we found that mRNA of pattern recognition receptors (PRRs), interferons (IFNs), and stimulated genes (ISGs) exhibited different levels of activation in the lungs and spleens of the two H5N6 viruses-inoculated geese though did not protect these birds from H5N6 HPAIVs infection. Our results suggested that the clade 2.3.4.4 waterfowl-origin H5N6 HPAIVs isolated from LPMs of Southern China could cause high mortality in geese and innate immune-related genes were involved in the geese innate immune response to H5N6 HPAIVs infection. Therefore, we should pay more attention to the evolution, pathogenic variations of these viruses and enhance virological surveillance of clade 2.3.4.4 H5N6 HPAIVs in waterfowls in China.

## 1. Introduction

Avian influenza viruses belong to family Orthomyxoviridae, genus Influenza A virus. Based on the antigenicity difference of the two surface proteins Hemagglutinin (HA) and Neuraminidase (NA), influenza A virus can be divided into 18 HA (H1-H18) and 11 NA (N1-N11) [1,2]. Wild waterfowls are the reservoirs of the influenza A viruses and most of the HA and NA subtypes are identified in these birds. Among these subtypes, H5 and H7 subtype viruses occasionally acquired a multibasic cleavage site insert in their HA genes, which always led to the emergence of highly pathogenic avian influenza viruses. To date, H5 and H7 subtype HPAIVs have widely circulated in China, posing great threat to poultry industries and public health [3,4,5].

In 1996, the first H5N1 HPAIV was detected in goose of Guangdong province, China [6]. Since then, H5N1 HPAIVs have continued to evolve into 10 different subclades (from clade 0 to 9) and spread through Asia to other continents including Europe, Africa, and North America, which have caused huge economic losses to the world poultry industry. More importantly, H5N1 HPAIVs have crossed the species barrier and infect human. The first report of human infection with H5N1 AIV was in Hong Kong of China, which caused 18 human infections in 1997 [7]. To date, there have been 861 cases of human infections with H5N1, which have posed great threat to public health [8]. Since 2008, clade 2.3.4.4 H5 AIVs (H5N2, H5N5, H5N6, and H5N8) have been increasingly detected in China [9,10,11,12]. Since 2014, there have been 24 cases of human infections with H5N6 viruses, posing a great threat to public health in China [8]. Recent study demonstrated that clade 2.3.4.4 H5N6 HPAIVs have become the dominant subtype in waterfowl in Southern China [13]. So far, the pathogenicity and transmission of the H5N6 HPAIVs in geese remain largely unclear.

The pathogenicity of AIV is associated with multiple factors, and both the viruses and host factors have contributed to the outcome of AIV infection [14]. Host innate immune response is crucial for protecting the host against influenza virus infection. However, influenza A viruses have developed many strategies to evade the host immune response. For example, NS1 of influenza A viruses is one of the well-studied IFNs antagonist proteins, which functions on multiple processes and inhibits the IFN-mediated antiviral response [15]. Therefore, it is important to understand the interaction of AIVs and hosts innate immunity. Previous researches have investigated the immune response of mouse, ferret, chicken, duck, and pigeon infected with H5N1 HPAIVs [16,17,18,19,20]. However, as one of the main hosts of H5N6 HPAIVs, the host innate immune response of geese infected with H5N6 HPAIVs have not been reported.

In this study, we have analyzed the genetic characteristic of two H5N6 viruses (GS38 and DK09) isolated from apparently healthy waterfowls in LPMs in Southern China 2016. We have further characterized the pathogenicity and transmission of the two H5N6 viruses in geese. In addition, we have quantified the mRNA of innate immune-related genes in the lungs and spleens of the H5N6 inoculated geese to provide useful data.

## 2. Materials and Methods

### 2.1. Viruses

The two avian influenza A (H5N6) viruses, A/goose/Guangdong/GS38/2016 (H5N6) and A/duck/Guangdong/DK09/2016 (H5N6) used in this study were isolated from apparently healthy domestic goose and duck in LPMs of Guangdong province, Southern China in 2016. The two H5N6 viruses were purified in 9 to 10 day-old specific-pathogen-free (SPF) embryonated chicken eggs, using methods described in the literature [21]. The 50% egg infective doses (EID_50_) was determined by infection of embryonated chicken eggs, values were calculated using the Reed-Muench method. The viruses were stored and frozen at −80 °C until use. All viral experiments were performed in Biosafety Level 3 (BSL-3) facilities.

### 2.2. Phylogenetic Analysis

Viral RNA was extracted from infectious allantoic fluid using Trizol LS Reagent (Life Technologies, Inc.) and reverse-transcribed to cDNA with the M-MLV reverse transcriptase (Promega) followed the manufacturer’s instruction. PCR amplification was performed using specific primers, and the PCR products were purified using a DNA Purification Kit (TIANGEN Biotech, China). The purified PCR products were sequenced by Shanghai Invitrogen Biotechnology Co., Ltd. The nucleotide sequences were analyzed and compiled using the Lasergene version 7. 1 (DNASTAR, USA). The phylogenetic trees of the two H5N6 viruses were constructed by distance-based neighbor-joining method with MEGA 5.0 software (Sinauer Associates, Inc., Sunderland, MA, USA). The reliability of these trees was evaluated with 1000 bootstrap replicates. The genetic distance and horizontal distances were proportional. The GenBank code of the reference sequences used for phylogenetic analysis are listed on Table S4. The nucleotide sequences obtained in this study are available from GenBank under the accession numbers (MT007264-MT007271; MT007272-MT007279)

### 2.3. Animal Infection Study

Four-week-old non-immune domestic geese (Qingyuan goose) were purchased from a farm near Guangzhou city and were housed in the ABSL-3 isolators. Geese were confirmed to be serologically negative for avian influenza with a hemagglutination inhibition (HI) test.

Inoculated groups (*n* = 15) were inoculated intranasally with 10^6^ EID_50_ of the GS38 and DK09 viruses in a volume of 0.2 mL, respectively. At 12 hours post-infection (HPI), three birds in each inoculated group were euthanized, and tissues (including liver, spleen, lung, kidney, intestine, pancreas, and bursa of Fabricius) were collected to detect the viral replication in geese. Contact group (*n* = 5) were co-housed with each inoculated group at 24 HPI to evaluate the transmission of the two H5N6 viruses in geese. At 3 days post-infection (DPI), three birds in each inoculated group were euthanized, and tissues (including liver, spleen, lungs, kidneys, intestine, pancreas, and bursa of Fabricius) were collected to detect the viral replication in geese. The collected lungs and spleens at 12 HPI and 3 DPI were also used to quantify the mRNA level of immune-related genes. The remaining inoculated geese (*n* = 9) were observed for clinical symptoms for 14 days or until all the geese were dead. All birds were labeled with wing ring to ensure individual identification. 

In addition, control group (*n* = 15) were only treated with 0.2 mL phosphate buffered saline (PBS). Three control geese were euthanized at 12 h and 3 days post-treatment, respectively. Tissues (including liver, spleen, lung, kidney, intestine, pancreas, and bursa of Fabricius) were collected to detect the viral replication in geese. The collected lungs and spleens at 12 h and 3 days post-treatment were also used to quantify the mRNA level of immune-related genes. The remaining control geese (*n* = 9) were observed for clinical symptoms for 14 days.

We also collected oropharyngeal and cloacal swabs from the inoculated and contact geese at 3, 5, 7, 9, 11, and 13 DPI to determine virus shedding. All collected samples were tested for viral replication by inoculated into SPF embryonated chicken eggs as described in the literature [21]. Reed-Muench method were used to calculate the viral titers.

### 2.4. Quantification of Innate Immune-Related Genes in H5N6 Infected Geese

To study the host innate immune response of geese infected with the H5N6 HPAIVs, we quantified the mRNA level of innate immune-related genes in the collected lungs and spleens of geese at 12 HPI and 3DPI. An Eastep®Super total RNA extraction kit (Promega, USA) was used to extract the total RNA from 50mg lungs and spleens of geese according to the manufacturer’s instruction. Total RNA (1 ug) was further reverse-transcribed to cDNA with the M-MLV reverse transcriptase (Promega) followed the manufacturer’s instruction, and the acquired cDNA was stored at −40 °C.

The primer pairs (Table 1) used for quantitative real-time PCR (qRT-PCR) were synthesized and selected according to specificity determined by dissociation curves and nucleotide sequencing. A GoTaq® qPCR master mix (Promega) was used to performed qRT-PCR experiment according to the manufacturer’s instruction. Bio-Rad CFX96 Touch™ Real-Time PCR Detection System (Bio-Rad Laboratories, USA) was used to perform the qRT-PCR with the following settings: 5 minutes at 95 °C, and then 15 seconds at 95 °C and 34 seconds at 62 °C for 40 cycles. Relative target gene expressions were calculated using the 2^–ΔΔCt^ method and expressed as a fold change in gene expression compared with control geese. GAPDH was used as the reference endogenous gene to normalize the quantification of the target gene.

### 2.5. Statistical Analysis

GraphPad Prism 7.0 software (GraphPad Software Inc, USA) was used to conduct statistical analysis. The Student’s t-test was used to analyze the statistical significance of the differences. A *p*-value < 0.05 was considered to be statistically significant (* *p* < 0.05; ** *p* < 0.01).

### 2.6. Ethics Statement

This study was carried out in ABSL-3 facilities in compliance with approved protocol (SCAUABSL2017-058) by the biosafety committee of South China Agriculture University. All animals experiment was handled in accordance with the principles of the Basel Declaration and recommendations of the approved guidelines of the Experimental Animal Administration and Ethics Committee of South China Agriculture University.

## 3. Results

### 3.1. Genetic Analysis of the Two H5N6 Viruses Isolated from Southern China in 2016

**Figure 1:**
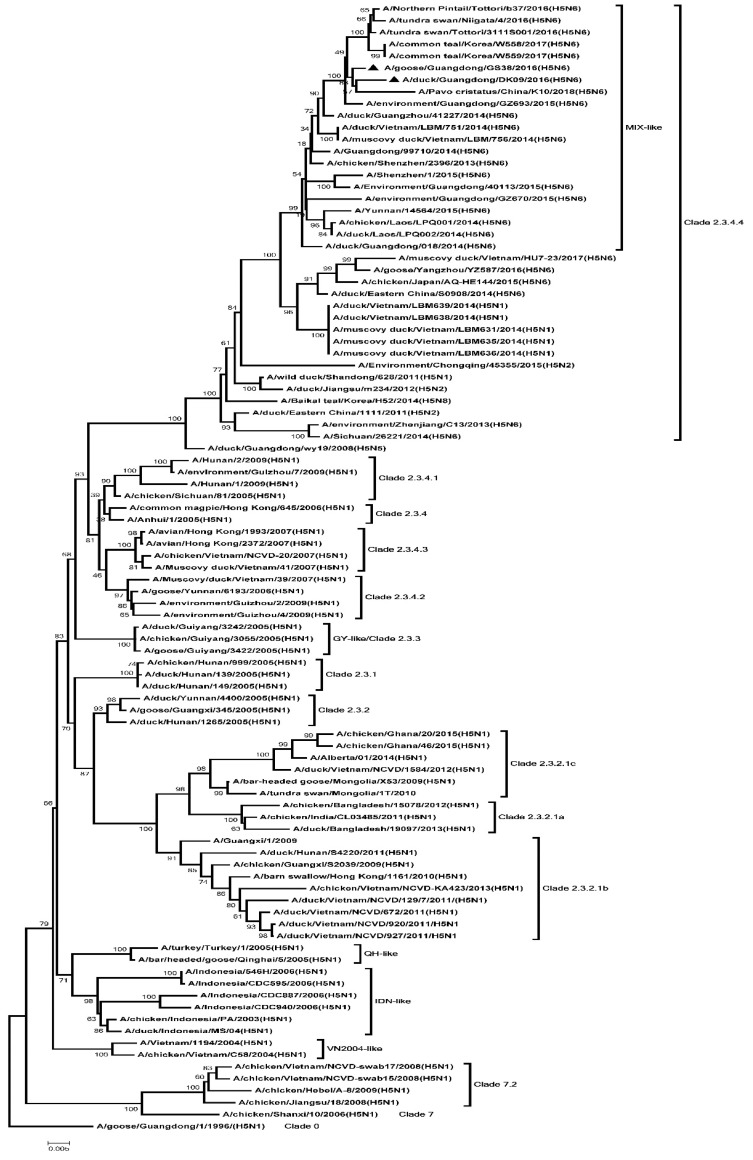
Phylogenetic analysis of Hemagglutinin (HA). Black triangles indicate viruses characterized in this study; other sequences were downloaded from GenBank. The two H5N6 viruses characterized in our study belonged to clade 2.3.4.4 and were clustered into the MIX-like group. QH, Qinghai; VN, Vietnam; IDN, Indonesia; GY, Guiyang; ZJ, Zhenjiang; MIX, viruses came from China, Japan, Vietnam, Korea, and so on.

To analyze the genetics of the two H5N6 viruses, eight genes of GS38 and DK09 viruses were sequenced. Results showed that the open reading frame (ORF) of the two H5N6 HA genes were 1701 bp, encoding 566 amino acids. The genetic analysis showed that HA genes of the two H5N6 viruses belonged to clade 2.3.4.4 and were clustered into the MIX-like group (Figure 1). The H5N6 viruses in MIX-like group have circulated in China, Japan, Korea, Laos, and Vietnam. Both the GS38 and DK09 H5N6 viruses possessed multiple basic amino acid residues (RERRRKR/GLF) at cleavage site of HA, which meets the characteristic of HPAIV (Table S1). The amino acid residues at positions of 226 and 228 of the two viruses were glutamine (Q) and glycine (G), respectively (H3 numbering system). Both the HA genes of the two H5N6 viruses contained seven potential N-linked glycosylation sites at 26 or 27, 39, 181, 302, 499, and 558, respectively (H5 numbering system) (Table S2).

**Figure 2:**
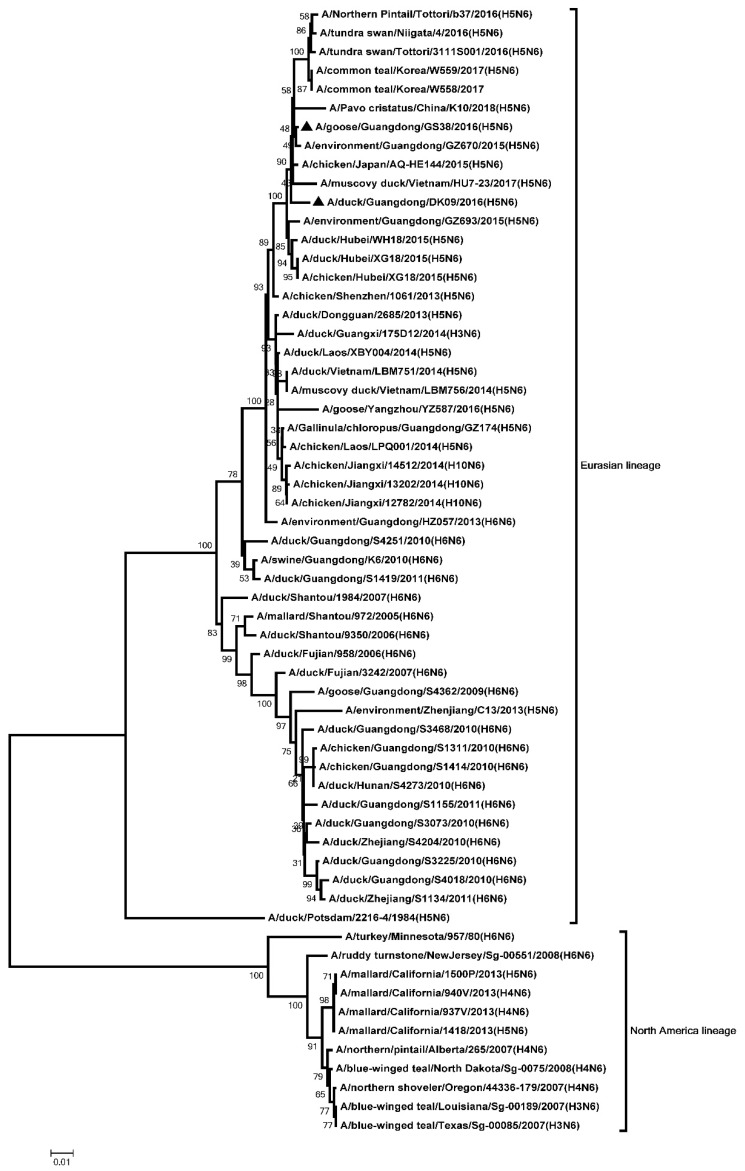
Phylogenetic analysis of Neuraminidase (NA). Black triangles indicates viruses characterized in this study, other sequences were downloaded from GenBank. The two H5N6 viruses characterized in our study were clustered into the Eurasian lineage.

The ORF of the two H5N6 NA genes were 1380 bp, encoding 459 amino acids. The genetic analysis showed that all the NA genes were classified into two groups: the Eurasia lineage and the American lineage. The NA genes of the two H5N6 viruses were clustered into the Eurasia lineage (Figure 2). An 11-amino acid deletion (58–68, N6 numbering system) was observed in the stalk of GS38 and DK09 NA genes (Table S1). Six potential N-linked glycosylation sites were present in NA gene of DK09 virus (51, 54, 70, 86, 146, and 201), and five potential N-linked glycosylation sites were observed in GS38 virus (51, 54, 70, 146, and 201) (Table S2).

**Figure 3:**
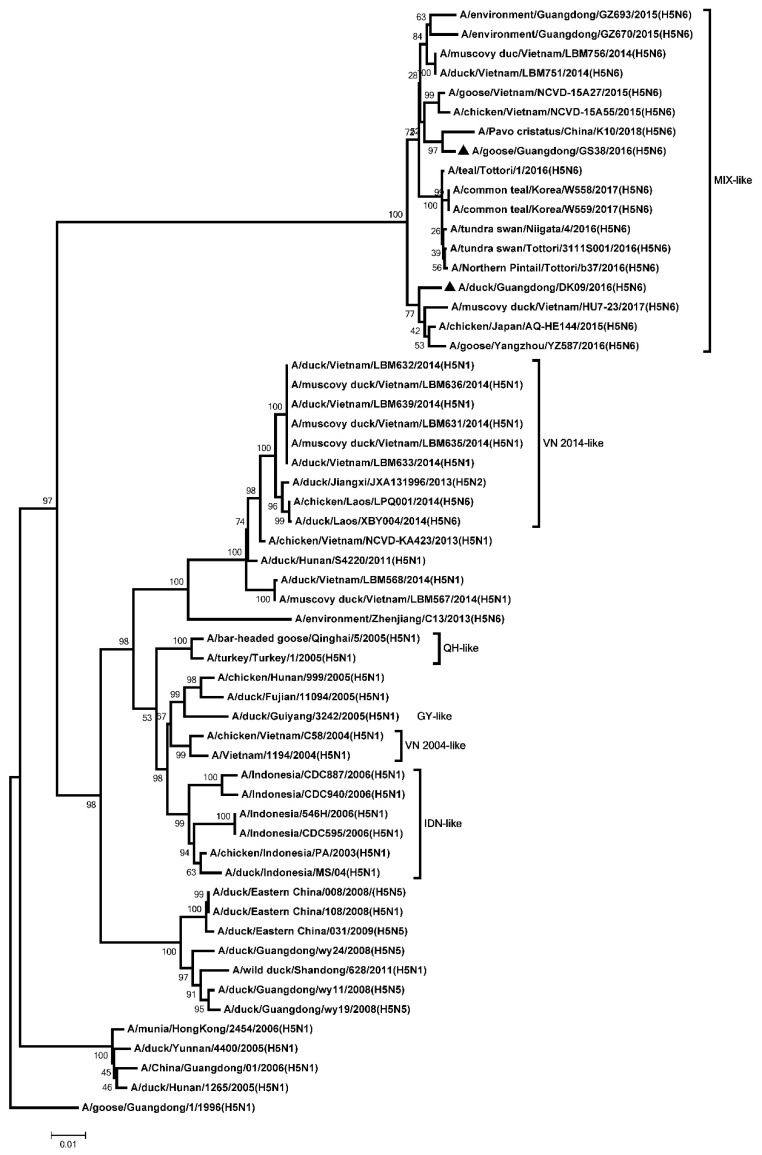
Phylogenetic analysis of PB2. Black triangles indicate viruses characterized in this study; other virus sequences were downloaded from GenBank. The two H5N6 viruses characterized in our study were clustered into the MIX-like group. QH, Qinghai; VN, Vietnam; IDN, Indonesia; GY, Guiyang; MIX, China, came from Japan, Vietnam, Korea and so on.

**Figure 4:**
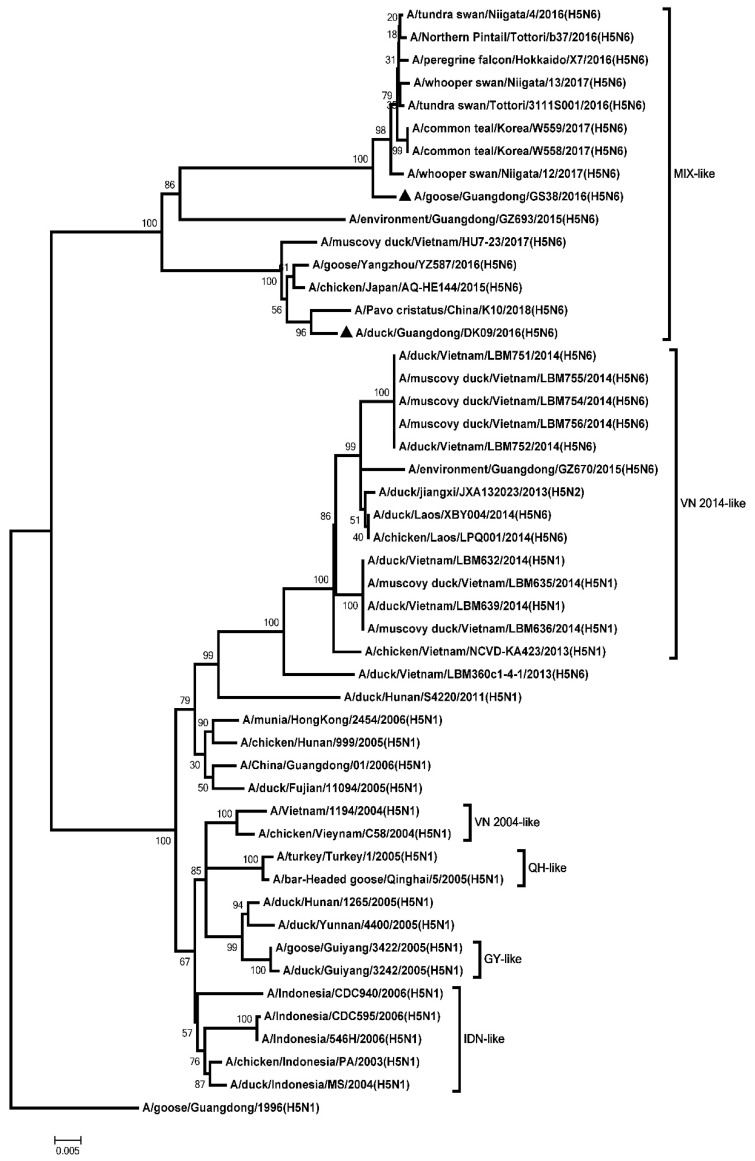
Phylogenetic analysis of PB1. Black triangles indicate viruses characterized in this study; other virus sequences were downloaded from GenBank. The two H5N6 viruses characterized in our study were clustered into the MIX-like group. QH, Qinghai; VN, Vietnam; IDN, Indonesia; GY, Guiyang; MIX, viruses came from China, Japan, Vietnam, Korea, and so on.

**Figure 5:**
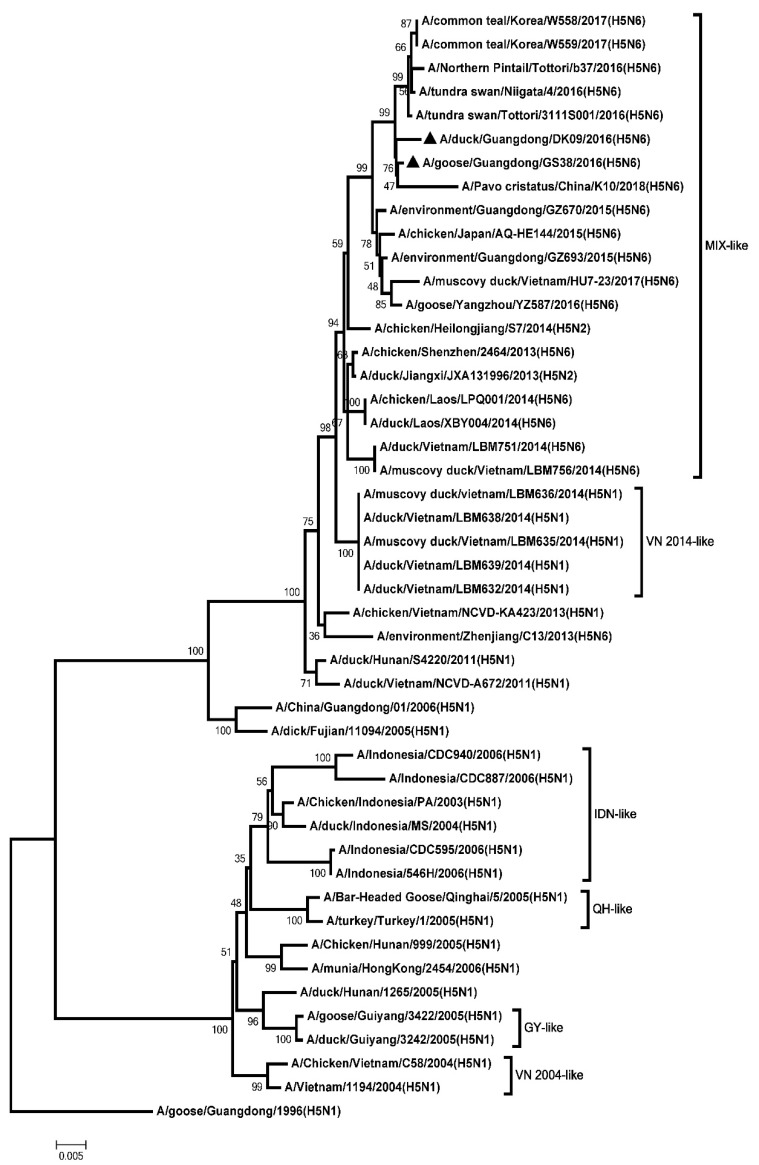
Phylogenetic analysis of PA. Black triangles indicates viruses characterized in this study, other virus sequences were downloaded from GenBank. The two H5N6 viruses characterized in our study were clustered into the MIX-like group. QH, Qinghai; VN, Vietnam; IDN, Indonesia; GY, Guiyang; MIX, viruses came from China, Japan, Vietnam, Korea, and so on.

**Figure 6:**
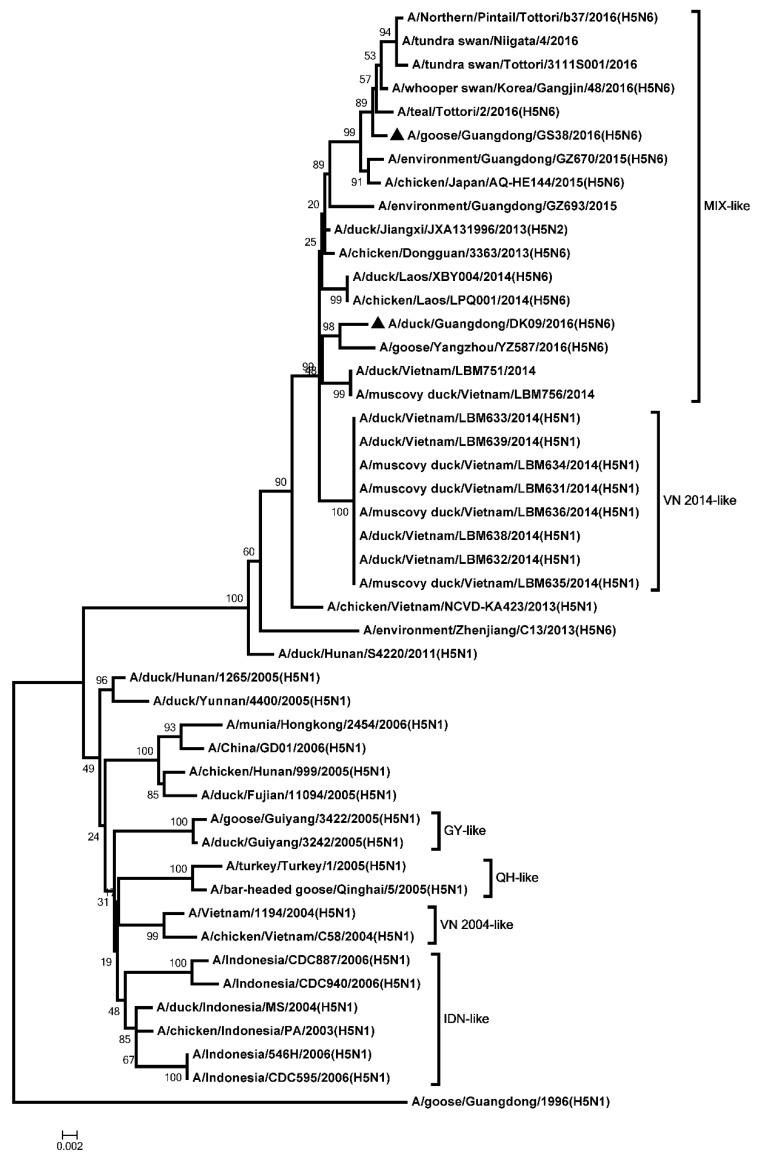
Phylogenetic analysis of NP. Black triangles indicate viruses characterized in this study; other virus sequences were downloaded from GenBank. The two H5N6 viruses characterized in our study were clustered into the MIX-like group. QH, Qinghai; VN, Vietnam; IDN, Indonesia; GY, Guiyang; MIX, viruses came from China, Japan, Vietnam, Korea, and so on.

**Figure 7:**
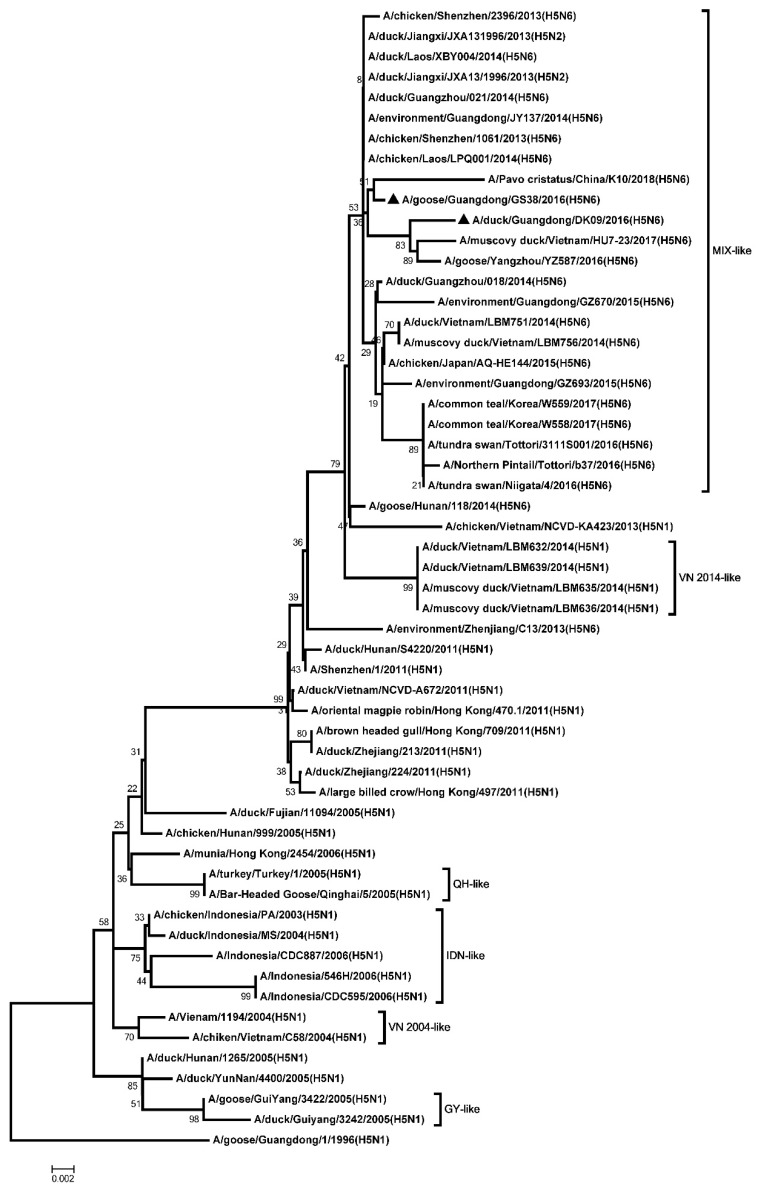
Phylogenetic analysis of M. Black triangles indicates viruses characterized in this study, other virus sequences were downloaded from GenBank. The two H5N6 viruses characterized in our study were clustered into the MIX-like group. QH, Qinghai; VN, Vietnam; IDN, Indonesia; GY, Guiyang; MIX, viruses came from China, Japan, Vietnam, Korea, and so on.

**Figure 8:**
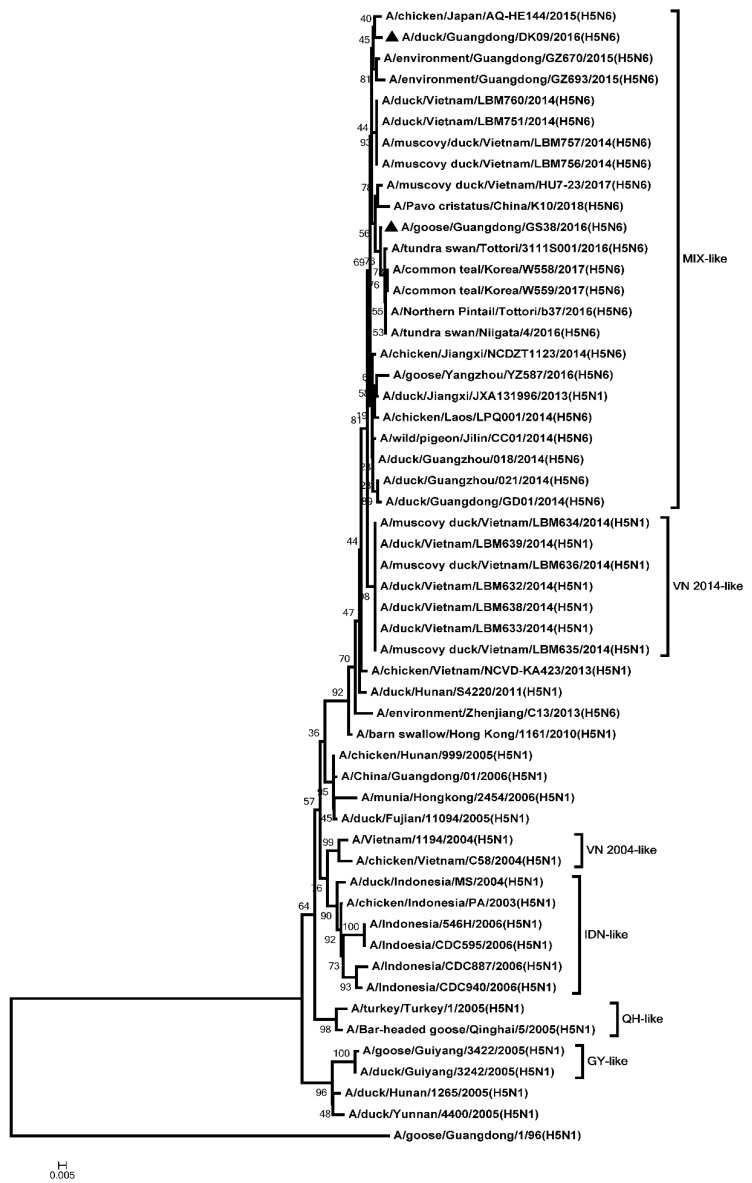
Phylogenetic analysis of NS. Black triangles indicate viruses characterized in this study; other virus sequences were downloaded from GenBank. The two H5N6 viruses characterized in our study were clustered into the MIX-like group. QH, Qinghai; VN, Vietnam; IDN, Indonesia; GY, Guiyang; MIX, viruses came from China, Japan, Vietnam, Korea, and so on.

The ORF of the PB2, PB1, PA, NP, M, and NS genes were 2280, 2274, 2151, 1497, 982, and 823 nucleotides, respectively. The six internal genes encode 8 proteins as follow: PB2, 759 amino acids; PB1, 757 amino acids; PA, 716 amino acids; NP, 498 amino acids; M1, 252 amino acids; M2, 97 amino acids; NS1, 225 amino acids; NS2, 121 amino acids. The genetic analysis showed that the PB2 genes of the two H5N6 viruses were classified into the MIX-like group (Figure 3). The amino acid residue at positions of 627 and 701 of the two viruses were glutamic (E) and aspartic acid (D), respectively (Table S1). The PB1 genes of the two H5N6 viruses were clustered into the MIX-like group (Figure 4). The amino acid residue at positions of 622 in PB1 at the two viruses is glycine acid (G) (Table S1). The genetic analysis showed that the PA genes of the two H5N6 viruses were classified into the MIX-like group (Figure 5). The NP genes of the two H5N6 viruses were clustered into the MIX-like group (Figure 6). The position of 184 amino acid residue in NP of the two viruses is lysine (K) (Table S1). The genetic analysis showed that the M genes of the two H5N6 viruses were classified into the MIX-like group (Figure 7). Mutations including L26F, A30V/T/S, S31N/G and G34E were not presented in the M2 genes of the two H5N6 viruses (Table S1). The NS genes of the two H5N6 viruses were clustered into the MIX-like group (Figure 8). Point mutations P42S and D92E were observed in the NS1 protein of the two H5N6 viruses (Table S1).

### 3.2. Pathogenicity, Replication, and Shedding of the Two H5N6 Viruses in Geese

To investigate the pathogenicity, replication and shedding of the two H5N6 viruses in geese, we inoculated geese with 10^6^ EID_50_ of the GS38 and DK09 viruses, respectively. Three of the inoculated geese were euthanized at 12 HPI and 3 DPI to investigate the viral replication in different tissues. Oropharyngeal and cloacal swabs from inoculated birds were collected at 3, 5, 7, 9, 11, and 13 DPI to determine the virus shedding in geese. All the remaining geese were observed daily for illness and death for 14 days.

Results showed that clinical symptoms began to appear in the two H5N6 HPAIVs inoculated geese at 3 DPI and lasted until 14 DPI. All GS38 and DK09 viruses inoculated geese exhibited serious clinical symptoms including severe neurologic signs, serious depression, anorexia, and green feces. GS38 virus killed 8/9 of the inoculated geese, with a mean dead time (MDT) of 6.4 DPI. DK09 virus killed 8/9 of the inoculated birds, with a MDT of 8.8 DPI (Table 2). Therefore, our results suggested that both the two H5N6 HPAIVs isolated from waterfowl in 2016, Southern China could cause high mortality in geese.

Additionally, we also tested the viral replication in the tissues of the GS38 and DK09 viruses inoculated geese. Results showed that the two viruses were replicated in all the tested tissues of inoculated geese, including liver, spleen, lungs, kidneys, brain, intestine, pancreas, and bursa of fabricius (Table 3). At 12 HPI, GS38 virus could be detected in 5 of 7 organs, with mean viral loads ranged from 1.58 to 2.92 log_10_EID_50_. DK09 virus could be detected in 6 of 7 organs, with mean viral loads ranged from 1.58 to 2.92 log_10_ EID_50_ (Table 3). At 3 DPI, GS38 virus was detected in all the tested tissues, with mean viral loads ranged from 1.83 to 4.17 log_10_EID_50_ DK09 virus was also detected in all the tested tissues, with mean viral loads ranged from 2.08 to 5.33 log_10_EID_50_. Therefore, our study demonstrated that both the GS38 and DK09 H5N6 viruses could replicate in multi-organs of inoculated geese.

The shedding of GS38 virus was detected in both oropharyngeal and cloacal swabs within 7 DPI (Table 4). DK09 virus was detected in oropharyngeal and cloacal swabs within 9 and 11 DPI, respectively. Notably, shedding of GS38 and DK09 viruses was detected in most of the inoculated geese at 5 and 7 DPI. (Table 4). Therefore, these results showed that geese could shed H5N6 viruses from respiratory and digestive tract after inoculated with the two viruses.

### 3.3. Transmission of the Two H5N6 Viruses in Geese

To determine the transmission of the GS38 and DK09 H5N6 viruses in geese, contact geese (*n* = 5) were co-housed with each inoculated group at 24 HPI. Both oropharyngeal and cloacal swabs from contact geese were collected at 3, 5, 7, 9, 11, and 13 DPI to test viral shedding. The contact geese were observed daily for illness and death until 14 DPI. 

Our results demonstrated that 4/5 of the GS38 virus contact geese showed obvious clinical symptoms and died, with a MDT of 8.8 DPI. The 3/5 of the DK09 virus contact geese exhibited obvious clinical symptoms and died, with a MDT of 12 DPI (Table 2). Both the two viruses were detected in the oropharyngeal and cloacal swabs from corresponding contact geese. The GS38 virus was detected in oropharyngeal swabs of contact geese at 3, 5, 7, and 9 DPI, and was detected in cloacal swabs at 3, 5 and 7 DPI. The DK09 virus was detected in both oropharyngeal and cloacal swabs at 5, 7, and 9 DPI (Table 4). Therefore, our results suggested that the GS38 and DK09 H5N6 viruses could transmit efficiently via direct contact between geese and were able to kill contact geese.

### 3.4. Quantification of PRRs, IFNs, and ISGs in the Lungs of the H5N6 Viruses Infected Geese

Lung is one of the most important target tissues for H5 subtype AIVs infection. To understand the expression of PRRs, IFNs, and ISGs in the lungs of H5N6 viruses infected geese, we quantified the mRNA of TLR7, TLR3, RIG-I, MDA5, IFN-α, IFN-γ, Mx and OASL in geese infected with GS38 and DK09 viruses at 12 HPI and 3 DPI.

**Figure 9:**
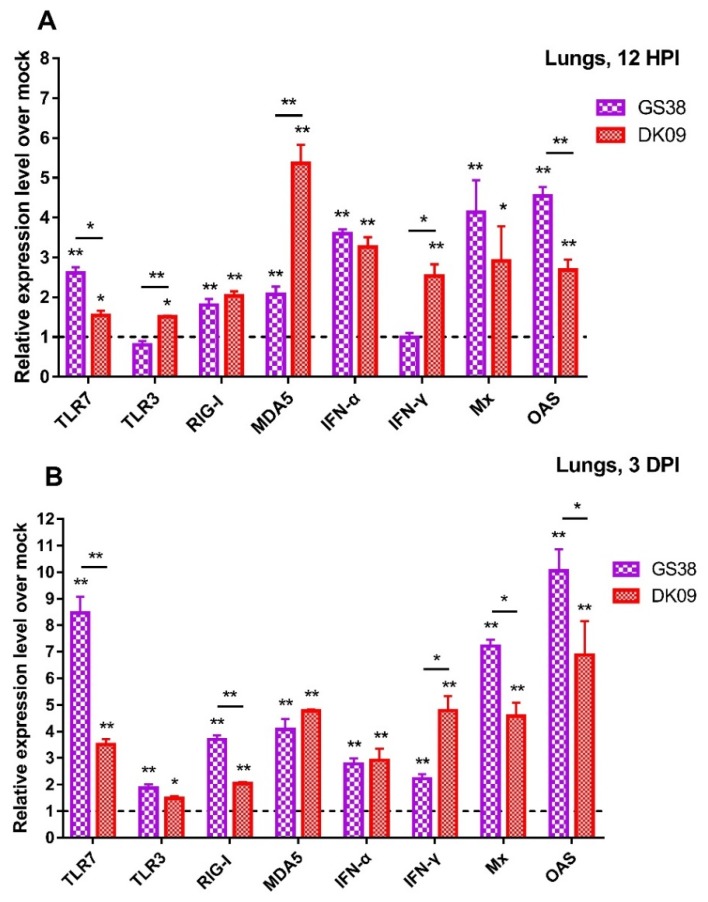
Relative expression of pattern recognition receptors (PRRs), interferons (IFNs), and stimulated genes (ISGs) in the lungs of geese inoculated with GS38 and DK09 viruses at 12 hours post-infection (HPI) (**A**) and 3 days post-infection (DPI) (**B**). The qRT-PCR was used to quantify the mRNA expression of TLR7, TLR3, RIG-I, MDA5, IFN-α, IFN-γ, Mx, and OASL in the lungs of H5N6 virus-infected geese. Each bar represents the level of target gene mRNA relative to mock after normalizing data to the housekeeping gene GAPDH and presented as the mean values ± standard deviation. The dotted line represents the mean level (*n* = 1) of the control group. Statistical analysis was performed using a Student’s t-test (* *p* < 0.05, ** *p* < 0.01).

At 12 HPI, the mRNA of TLR7, RIG-I and MDA5 were significantly upregulated (1.54 to 5.36 fold, *p* < 0.05) in the two H5N6 viruses-inoculated geese compared to that of control geese (Figure 9A). The mRNA of TLR3 in response to DK09 was increased (1.50 fold, *p* < 0.05) yet slightly decreased in response to GS38 compared to that of control geese. The expression of IFN-α, Mx and OASL exhibited different levels of activation in the two H5N6 viruses-inoculated geese (2.68 to 4.54 fold, *p* < 0.05) over that of control geese. The mRNA of IFN-γ was significantly upregulated in DK09 infected geese (2.53 fold, *p* < 0.05) but hardly change in GS38 infected geese (Figure 9A).

At 3 DPI, the expression of TLR7, TLR3, RIG-I and MDA5 showed different level of upregulation (1.48 to 8.46 fold, *p* < 0.05) in the two H5N6 viruses infected geese over that of control (Figure 9B). Interestingly, the TLR7 mRNA was significantly activated in response to GS38 virus (8.46 fold, *p* < 0.01). The mRNA of IFN-α, Mx and OASL in the two viruses inoculated geese exhibited higher level of upregulation (2.23 to 10.05 fold, *p* < 0.05) compared to those of control geese. Notably, the expression of Mx and OASL was significantly increased in response to the two H5N6 viruses (4.58 to 10.05 fold, *p* < 0.01) compared to that of control geese (Figure 9B). 

### 3.5. Quantification of PRRs, IFNs, and ISGs in the Spleens of H5N6 Viruses Infected Geese

Spleen is the largest immune organs in goose and plays an important role in protecting host against viral infection. To investigate the mRNA of PRRs, IFNs and ISGs in the spleens of H5N6 viruses infected geese, we quantified the mRNA expression of TLR7, TLR3, RIG-I, MDA5, IFN-α, IFN-γ, Mx, and OASL at 12 hours and 3 days post-infection with the two H5N6 viruses.

**Figure 10:**
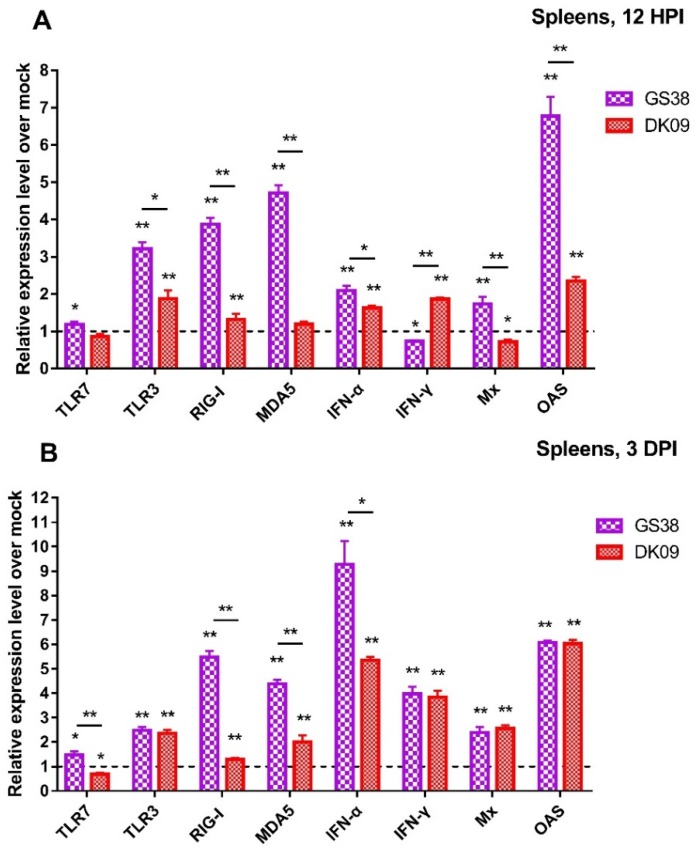
Relative expression of PRRs, IFNs, and ISGs in the lungs of geese inoculated with GS38 and DK09 viruses at 12 HPI (**A**) and 3 DPI (**B**). The qRT-PCR was used to quantify the mRNA expression of TLR7, TLR3, RIG-I, MDA5, IFN-α, IFN-γ, Mx, and OASL in the spleens of H5N6 virus-infected geese. Each bar represents the level of target gene mRNA relative to mock after normalizing data to the housekeeping gene GAPDH and presented as the mean values ± standard deviation. The dotted line represents the mean level (*n* =1) of the control group. Statistical analysis was performed using a Student’s t-test (* *p* < 0.05, ** *p* < 0.01).

At 12 HPI, TLR7 mRNA was slightly changed in response to GS38 and DK09 viruses infection. The mRNA of TLR3, RIG-I, and MDA5 in GS38 inoculated geese exhibited higher activation (3.21 to 4.71 fold, *p* < 0.01) compared to those of control (Figure 10A). The expression of TLR3 and RIG-I was slightly upregulated in response to DK09 virus (1.88 and 1.32 fold, respectively, *p* < 0.01). The mRNA of IFN-α and OASL in the two H5N6 viruses inoculated geese were significantly activated (1.63 to 6.77 fold, *p* < 0.05) compared to those of control geese. The expression of IFN-γ was upregulated in response to DK09 virus (1.87 fold, *p* < 0.05) but downregulated in response to GS38 virus (0.73 fold, *p* < 0.05). The Mx mRNA also exhibited a different expression level in response to the two viruses.

At 3 DPI, TLR7 mRNA was slightly upregulated in response to GS38 virus (1.48 fold, *p* < 0.05) and significantly downregulated in response to DK09 virus (0.68 fold, *p* < 0.05) (Figure 10B). The mRNA of TLR3, RIG-I and MDA5 in the two viruses inoculated geese exhibited different activation level (1.30 to 5.48 fold, *p* < 0.05) compared to those of control geese. Interestingly, mRNA of RIG-I and MDA5 showed a higher activation in response to GS38 virus (4.38 and 5.48 fold, respectively, *p* < 0.01). The mRNA of IFN-α, IFN-γ, Mx, and OASL in the two viruses inoculated geese were significantly upregulated (2.39 to 9.27 fold, *p* < 0.01) compared to those of control geese (Figure 10B).

## 4. Discussion

Waterfowls are natural hosts of AIVs, they usually show asymptomatic infection after infected with AIVs, which is helpful for circulation and spread of AIVs [22]. However, since 2003, some H5N1 HPAIVs were lethal to waterfowls and caused great losses to waterfowl industry [23]. Since 2014, clade 2.3.4.4 H5 HPAIVs combined with different NA subtypes (N2, N5, N6, and N8) were identified and circulated in China, Japan, Korea, Laos, and Vietnam. Among these viruses, H5N6 HPAIVs has crossed the species barrier and caused human infection cases, posing a new threat to public health in China. Notably, among these human infection cases, most of the patients have had a contact history with the infected poultry in live poultry markets (LPMs) or backyard poultry flocks (BPFs), which are considered the major risk factors in human infection cases [24,25]. Recent epidemiological study demonstrated that H5N6 HPAIVs have replaced H5N1 HPAIVs as the dominant subtype in waterfowls in Southern China. From 2018 to Jan 2020, H5N6 HPAIVs have circulated in poultry and caused outbreaks in China, Vietnam, Japan, North Korea, and the Netherlands, etc., caused huge losses to the poultry industry. Among these countries, 11 outbreaks of poultry infection with H5N6 HPAIVs were report from China, which led to death and culling of over 214,651 birds [26]. More importantly, since 2014, there have been 24 cases of human infections with H5N6 viruses, posing a great threat to public health in China. Therefore, it is necessary to understand the genetics, pathogenicity and transmission characteristic of these H5N6 viruses derived from waterfowls in LPMs of Southern China.

In this study, we isolated two H5N6 viruses (GS38 and DK09) from swabs of apparently healthy waterfowls in LPMs in Guangdong, China, 2016. The HA genes of the two H5N6 viruses belonged to clade 2.3.4.4 and were further classified into the MIX-like group, which circulated around China, Japan, Korea and Vietnam. Both the GS38 and DK09 H5N6 viruses possessed multiple basic amino acid residues (RERRRKR/GLF) at cleavage site of HA, which suggested that both of them are HPAIVs [27]. Typically, avian and human influenza viruses have a different α2-3- and α2-6-linked sialic acid binding preference [28]. However, previous studies have demonstrated that some amino acid mutations in HA genes might lead to binding preference switch from α2-3 “avian-type” receptors to α2-6 “human-type” receptors, which was regarded as an important factor for pandemic potential of these viruses. Point mutations Q226L and G228S in HA genes were the well-studied amino acid mutations that contributed to the H5N1 AIVs binding preference change [29]. The two-point mutations were not observed in the two H5N6 HPAIVs. However, other mutations including S137A, S158N and T160A were observed in the two H5N6 viruses, which have been associated with increased binding to “human-type” receptors [30,31,32]. Therefore, we should pay more attention to whether these mutations influence the receptor-binding preference of the clade 2.3.4.4 H5N6 HPAIVs. NA is one of the major glycoproteins in the surface of AIV, which mediate the release of progeny virions from the infected cells and facilitates virus spread within the respiratory tract. The NA genes of the two H5N6 viruses were classified into the Eurasian lineage, which derived from H6 subtype AIVs. An 11-amino acid deletion (58–68, N6 numbering) was detected in the stalk of NA genes, which might enhance virulence toward mammal [33]. Mutations including V116A, E119A, H275Y, R293K, and N295S (N6 numbering) in NA genes have been associated with reduced susceptibility to neuraminidase inhibitors [34,35,36]. None of these mutations was observed in the NA genes of the two H5N6 viruses, which suggested that the two H5N6 viruses were sensitive to oseltamivir and zanamivir.

The internal genes including PB2, PB1, PA, NP, M, and NS of the two H5N6 viruses derived from the MIX-like. Mutation E627K is the best-known point mutation in the PB2 gene, which is considered associated with increased virulence of H5N1 AIVs in mammalian model [37]. Other mutations in PB2 genes like E192K, Q591K, and D701N were also associated with mammalian adaptation [38,39,40,41]. None of these mutations was observed in the PB2 genes of the two H5N6 viruses. Point mutation D622G was observed in the PB1 genes of the two H5N6 viruses, which was demonstrated to be associated with increased polymerase activity and virulence in mice [42]. Point mutation A184K was observed in the NP genes of the two H5N6 viruses, which might result in increased virulence in chickens. Mutations including L26F, A30V/T/S, S31N/G, and G34E were not presented in the M2 protein of the two H5N6 viruses, which suggested that the two H5N6 viruses were sensitive to amantadine and rimantadine [43]. The NS1 has been reported to be the major viral antagonist of the host antiviral response in mice and pigs. However, point mutations P42S and D92E were observed in the NS1 protein of the two H5N6 viruses, which may decrease the host IFN response in mammalian model [44,45].

Therefore, our results suggested that the two H5N6 viruses belonged to clade 2.3.4.4 and were reassortants of the H5N1 and H6N6 viruses and likely derived from the same ancestor. Some characterized mutations were observed in these two viruses, which suggested that continued surveillance of these H5N6 viruses in LPMs is a necessity.

In our previous studies, we characterized the pathogenicity and transmission of four different clade H5N1 HPAIVs in geese. Results show that A/Duck/Guangdong/212/2004 (clade 9) killed 2/5 of inoculated geese; A/Duck/Guangdong/E35/2012 (clade 2.3.2.1) killed 1/3 of inoculated geese; A/Goose/Guangdong/1/1996 (clades 0) and A/Chicken/Henan/B30/2012 (clade 7.2) did not kill the inoculated geese. Furthermore, A/Duck/Guangdong/212/2004 (clade 9) killed 1/3 of contact geese; the other three viruses could not transmit from inoculated geese to contact geese [46,47]. Recent study have shown that clade 2.3.4.4 H5N1 HPAIVs isolated from Canada was moderately pathogenic to Chinese geese [48]. Above studies suggested that H5N1 HPAIVs derived from different subclades exhibited different pathogenicity and transmission in geese. In this study, clade 2.3.4.4 H5N6 HPAIVs caused serious illness in inoculated geese and with high mortality in these birds. The GS38 and DK09 viruses killed 8/9 and 8/9 of the inoculated geese, respectively. The two viruses were detected in both respiratory and digestive tract, which indicated that the infected geese could shed H5N6 viruses efficiently. Furthermore, GS38 and DK09 viruses killed 4/5 and 3/5 of the contact geese, respectively. Both the two viruses contact geese could shed viruses from respiratory and digestive tract. In this study, we only tested the shedding of the viruses in oropharyngeal and cloacal swabs, did not quantify the exact viral titers in the swabs. The mean dead time of GS38 virus inoculated and cohoused geese were 6.4 and 8.8 days post-infection, respectively. While the mean dead time of DK09 virus inoculated and cohoused geese were 10.4 and 12 days post-infection, respectively. Some mutations between the two viruses might be attributed to these results. There were 47 amino acid residues differences between the two viruses (10 in PB2, 15 in PB1, 2 in PA, 4 in HA, 3 in NP, 7 in NA, 2 in M2 and 4 in NS1). We have listed and analyzed the potential virulence determinants in the HA, NA, PB2 and NS1 genes of the two viruses in Table S3. Among these mutations, I292V and A588V in PB2 genes have been reported associated with increased polymerase activity in mammalian and/or avian cell line and increased virulence in mice [49,50]. The two mutations were observed in GS38 virus but not DK09 virus, which might contribute to the difference of the mean death time. Our results demonstrated that this clade 2.3.4.4 H5N6 HPAIVs isolated from Southern China in 2016 could cause a high mortality in geese and be transmitted more efficiently in these birds compared with previous reports. Furthermore, our previous studies suggested that chicken-origin and goose-origin H5N6 HPAIVs isolated from Southern China in 2016 exhibited high pathogenicity in chickens [51]. In addition, duck-origin and goose-origin H5N6 HPAIVs were also able to kill ducks [52]. Therefore, these results suggested that clade 2.3.4.4 H5N6 HPAIVs isolated from Southern China in 2016 have become a huge threat to poultry industry.

Innate immunity is the first line of defense against pathogens. Upon viral infection, host pattern recognition receptors (PRRs) recognize the conserved viral component called pathogen-associated molecular patterns (PAMPs) to activate the innate immune signaling pathway. In mammals, Toll-like and RIG-I like receptors are the most important PRRs in detecting influenza viruses infection and triggering host innate immune response. However, there is limited knowledge about the host innate immune response of goose to AIV infection. Our previous studies have identified TLR7 and MDA5 in goose and found that mRNA of goose TLR7 and MDA5 were upregulated in the lungs of H5N1 infected geese [47,53]. In this study, expression of TLR7 and MDA5 was also observed an upregulation in the lungs of H5N6 infected geese, though with different activation level in response to the two H5N6 viruses. Waterfowls are natural host of AIVs and usually considered highly resistant to influenza virus infections. RIG-I is absent in chickens but present in ducks, which was considered an essential factor of natural resistance to the influenza virus in ducks [54]. Our previous studies have demonstrated that duck RIG-I was significantly increased in the lungs of H5N1 infected ducks [19]. Goose also possesses RIG-I and was considered to functions in innate immunity against NDV infections [55]. In this study, the expression of RIG-I was upregulated in the lungs and spleens after inoculated with the two H5N6 HPAIVs, though with varying activation level in the two organs. Therefore, our study demonstrated that goose Toll-like and RIG-I like receptors were involved in host immune response at the early stage of H5N6 HPAIVs infection.

Upon the recognition of PAMPs, PRRs-mediated signal pathway could induces the expression of type I interferons (IFNs). The secretion of IFNs could enhance the expression of ISGs and pro-inflammatory cytokines via the JAK/STAT pathway to limit viral replication. Type I IFN(α/β) signal pathway have been well studied in mammals, which considered playing a central role in the innate antiviral immunity, but related knowledge on birds is relatively limited. Goose IFN-β has not been characterized so far. Previous studies have demonstrated that goose IFNs (IFN-α, IFN-γ, and IFN-λ) could induce the expression of Mx and OASL, which established antiviral state against duck Tembusu virus (TMUV) infection in vitro [56]. The mRNA of IFN-α, IFN-γ, Mx, and OASL were also observed a significantly upregulation in the spleens of geese infected with TMUV [57]. Above studies suggested that goose IFN-mediated signal pathway might function in the immune response to TMUV infection. In our previous studies, though the upregulation of OASL and PKR was observed in the lungs of the H5N1 infected geese, these cytokines did not protect the geese from H5N1 HPAIV infection [47]. In other reports, H5N1 HPAIV also induced high activation of IFNs response in lungs of infected chickens, but no infected chickens survived. Notably, treatment with recombinant chicken IFN-α also failed to confer protection against H5N1 HPAIV challenge in chicken [58]. Similarly, a significant upregulation of IFNs (α/β/γ) was induced in the lungs of H5N6 infected chickens, but none of these infected chickens survived [59]. In this study, the expression of IFN-α, IFN-γ, Mx, and OASL was also upregulated in the lungs and spleens of H5N6 inoculated geese, but the viral titers continued to increase in these organs and most of the inoculated geese died within 14 days. Therefore, above studies suggested that the induced innate immune-related molecules such as IFNs, Mx, and OASL might not be sufficient to counteract those H5 subtype AIVs that are highly pathogenic to their hosts. However, we did not quantify the mRNA of immune-related genes in the survived geese, so it isn’t clear if the survivals from each group had different activation levels of innate immune response genes compared to the ones which later succumbed to infection.

Furthermore, we found that geese infected with the two viruses exhibited different activation level of the tested immune-related genes in the lungs and spleens. In the lungs, geese infected with GS38 virus exhibited higher expression of TLR7, RIG-I, Mx, and OASL at 3DPI than those infected with DK09. DK09 virus induced higher expression of TLR3, MDA5, and IFN-γ in the lungs than GS38 at 12 HPI (Figure 9). In the spleens, GS38 virus induced a higher expression of TLR3, RIG-I, MDA5, IFN-α, Mx, and OASL than DK09 virus at 12 HPI. In addition, GS38 virus induced a higher expression of TLR7, RIG-I, MDA5, and IFN-α at 3 DPI than DK09 (Figure 10). Above results suggested that immune-related genes were involved in the immune response to the two viruses’ infection though with different activation level. The pathogenicity of AIV is associated with multiple factors, and both the viruses and host factors have contributed to the outcome of AIV infection [14]. In this study, though the two viruses to some extent induced different innate immune response in the infected geese, no significant difference of pathogenicity or viral replication were observed between the two viruses (Table 2 and Table 3). These results suggested that the activation levels of the immune-related genes induced by influenza viruses might be not related to their pathogenicity in geese.

In summary, our results showed that clade 2.3.4.4 H5N6 HPAIVs (GS38 and DK09) isolated from LMPs of Southern China were able to cause high mortality in geese and were transmitted efficiently between these birds, which indicated that these viruses might become a new threat to the poultry industry and public health. Furthermore, we found that immune-related genes (PRRs, IFNs, and ISGs) were involved in the geese immune response to H5N6 AIVs infection, but the activated host immune response could not protect geese from H5N6 HPAIVs infection. Therefore, it is necessary to conduct continued surveillance of H5N6 HPAIVs in the waterfowls in Southern China.

## Figures and Tables

**Table 1:** Primer sequences used in the qRT-PCR.

Genes	Forward Primer Sequences (5′–3′)	Reverse Primer Sequences (5′–3′)	Accession No.
TLR7	CCTTGTATTCCCTGCCCCAA	ACTTCAAAAACTGAGCATCT	KJ022638
TLR3	CGGTCGGAAAGCAATGTCAA	AGCTGATTATGAGAAATGTC	KP238287
RIG-I	AGCACCTGACAGCCAAAT	AGTGCGAGTCTGTGGGTT	JF804977
MDA5	TGCTGTAGTGGAGGATTTG	CTGCTCTGTCCCAGGTTT	JX976550
IFN-α	CAGCACCACATCCACCAC	TACTTGTTGATGCCGAGGT	AY524422
IFN-γ	TGAGCCAGATTGTTTCCC	CAGGTCCACGAGGTCTTT	AY524421
Mx	TTCACAGCAATGGAAAGGGA	ATTAGTGTCGGGTCTGGGA	KU247604
OASL	CTGGCGGTGAAGAATACGGT	CGTCTTGAAGACGCGGATTT	KU058695
GAPDH	CATCTTCCAGGAGCGCGACC	AGACACCGGTGGACTCCACA	MG674174

**Table 2:** Mortality of geese after inoculated with H5N6 highly pathogenic avian influenza viruses.

Strains	Group	Illness ^a^	Death	MDT
GS38	Inoculated ^b^	9/9	8/9	6.4
	Contact ^c^	4/5	4/5	8.8
DK09	Inoculated	9/9	8/9	10.4
	Contact	3/5	3/5	12

Abbreviations: MDT, mean dead time. a: The data show the number of dead and the total number of birds from the observation period. b: Geese inoculated with virus. c: Naive contact geese housed with those inoculated geese.

**Table 3:** Replication of H5N6 highly pathogenic avian influenza viruses in geese.

Strains	Time	Virus Replication Titers in Organs of Inoculated Geese (log_10_EID_50_/0.1 mL) ^a^						
		Liver	Spleen	Lung	Kidney	Intestine	Pancreas	Bursa of Fabricius
GS38	12HPI	2.92 ± 0.58	1.58 ± 0.14	2.25 ± 0.75	< 1.5	1.58 ± 0.14	1.75 ± 0.43	< 1.5
	3 DPI	1.83 ± 0.58	4.17 ± 1.53	2.83 ± 1.28	3.92 ± 1.51	3.75 ± 1.98	2.83 ± 1.89	3.67 ± 1.23
DK09	12HPI	1.58 ± 0.14	<1.5	2.17 ± 1.15	2.33 ± 1.44	2.92 ± 0.38	1.92 ± 1.42	2 ± 0.87
	3 DPI	4.75 ± 2.95	3.92 ± 3.17	5.33 ± 1.80	2.08 ± 0.52	3.17 ± 2.89	2.75 ± 2.15	3.33 ± 1.67

Abbreviations: HPI, hour post-inoculation; DPI, day post-inoculation; a: For statistical analysis, a value of 1.5 was assigned if the virus was not detected from the undiluted sample in three embryonated hen eggs. Viral loads were expressed as mean ± SD in log_10_EID_50_/0.1 mL of tissue.

**Table 4:** Viral shedding in cloacal and oropharyngeal swabs.

Strain	Infection Sample	3 DPI		5 DPI		7 DPI		9 DPI		11 DPI		13 DPI	
		T	C	T	C	T	C	T	C	T	C	T	C
GS38	Inoculated ^a^	2/12	1/12	5/6	5/6	3/3	2/3	0/1	0/1	0/1	0/1	0/1	0/1
	Contacted ^b^	1/5	1/5	4/5	3/5	2/3	2/3	1/3	0/3	0/2	0/2	0/1	0/1
DK09	Inoculated	2/12	1/12	6/9	2/9	5/7	6/7	2/6	2/6	0/5	2/5	0/1	0/1
	Contacted	0/5	0/5	1/5	1/5	2/5	1/5	3/5	2/5	0/4	0/4	0/2	0/2

Abbreviations: DPI, day post-inoculation; T, oropharyngeal swab; C, cloacal swab. a: Geese inoculated with virus. b: Naive contact geese housed with those inoculated.

## Supplementary Materials

The following are available online at https://www.mdpi.com/2076-2607/8/2/224/s1. Table S1: Molecular analysis of important amino acid residues in the genome of the two H5N6 HPAIVs; Table S2: List of potential glycosylation site in HA and NA genes of the GS38 and DK09 viruses; Table S3: Amino acid residues differences in HA, NA, PB2 and NS1 between GS38 and DK09 H5N6 HPAIVs; Table S4: Genebank code of the reference sequences used in Figure 1, Figure 2, Figure 3, Figure 4, Figure 5, Figure 6, Figure 7 and Figure 8.
