# Supplementary material for: Host Innate Immune Response of Geese Infected with Clade 2.3.4.4 H5N6 Highly Pathogenic Avian Influenza Viruses
Source: Microorganisms. 2020 Feb 7;8(2):224. doi: 10.3390/microorganisms8020224 (PMC7074872; doi:10.3390/microorganisms8020224)
Supplement: Supplementary file 1 [file microorganisms-08-00224-s001.pdf]

microorganisms

MDPI

Supplementary Materials

Table S1. Molecular analysis of important amino acid residues in the genome of the two H5N6 HPAIVs.

Viral proteins	Phenotype	Mutations	GS38	DK09
HA[a]	Increased virus binding to α2–6	S137A	A	A
		S158N	N	N
		T160A	A	A
		E190G	E	E
		N197K	N	N
		V214I	V	V
		Q226L	Q	Q
		G228S	G	G
	Cleavage site	-RRRKR ↓ G-	Yes	Yes
NA[b]	Increased virulence in mice	58-68 Stalk deletion	Yes	Yes
	Reduced susceptibility to oseltamivir and zanamivir	E119A	E	E
		H275Y	H	H
		R293K	R	R
		N295S	N	N
PB2[c]	Mammalian adaptation	E192K	E	E
		Q591K	Q	Q
		E627K	E	E
		D701N	D	D
PB1[c]	Increased polymerase activity and virulence in mice	N105S	N	N
		D622G	G	G
PA[C]	Increased polymerase activity and virulence in mice	T97I	T	T
NP[c]	Increased virulence in chickens	M105V	I	I
		I109T	I	I
		A184K	K	K
NS1[Cc]	Increased virulence in mice	P42S	S	S
		D92E	E	E
M2[c]	Increased resistance to amantadine and rimantadine	L26F	L	L
		A30V/T/S	A	A
		S31N/G	S	S
		G34E	G	G

a H3 numbering system is used. b N6 numbering system is used. c H5 numbering system is used.

Table S2. List of potential glycosylation site in HA and NA genes of the GS38 and DK09 viruses.

Genes	Position	Viruses	
		**GS38**	**DK09**
HA[a]	26	NNS	NNS
	27	NST	NST
	39	NVT	NVT
	181	NNT	NNT
	302	NSS	NSS
	499	NGT	NGT
	558	NGS	NGS
NA[b]	51	NET	NET
	54	NPT	NPT
	70	NIT	NIT
	86	-[c]	NLT
	146	NGT	NGT
	201	NAS	NAS

[a] H5 numbering system is used. [b] N6 numbering system is used. [c] The potential N-glycosylation sites at position is absent.

Table S3. Amino acid residues differences in HA, NA, PB2 and NS1 between GS38 and DK09 H5N6 HPAIVs.

Genes	Position	Viruses	
		**GS38**	**DK09**
HA	51	R	K
	56	K	R
	146	V	M
	337	L	S
NA	50	I	M
	59	N	K
	75	K	N
	251	K	R
	310	K	R
	331	T	I
	430	T	A
PB2	152	T	A
	178	A	T
	292	V	I
	398	I	L
	441	N	D
	448	N	T
	456	S	N
	495	V	L
	559	S	N
	588	V	I
NS1	134	N	D
	147	E	K
	221	I	V
	225	I	V

Table S4. Genebank code of the reference sequences used in Figure 1–8.

Strains	Genebank code of reference sequences							
	PB2	PB1	PA	HA	NP	NA	M	NS
A/Northern Pintail/Tottori/b37/2016(H5N6)	LC200414	LC200415	LC200416	LC200417	LC200418	LC200419	LC200420	LC200421
A/tundra swan/Niigata/4/2016(H5N6)	LC316704	LC316705	LC316706	LC316707	LC316708	LC316709	LC316710	LC316711
A/tundra swan/Tottori/3111S001/2016(H5N6)	LC274915	LC274916	LC274917	LC274918	LC274919	LC274920	LC274921	LC274922
A/common teal/Korea/W558/2017(H5N6)	KY576130	KY576128	KY576126	KY576116	KY576122	KY576120	KY576118	KY576124
A/common teal/Korea/W559/2017(H5N6)	KY576131	KY576129	KY576127	KY576117	KY576123	KY576121	KY576119	KY576125
A/Pavo cristatus/China/K10/2018(H5N6)	MK027384	MK027385	MK027386	MK027387	MK027388	MK027389	MK027390	MK027391
A/environment/Guangdong/GZ693/2015	KU852959	KU852956	KU852953	KU852944	KU852950	KU852947	KU852962	KU852966
A/duck/Guangzhou/41227/2014(H5N6)	KP765793	KP765794	KP765795	KP765796	KP765797	KP765798	KP765799	KP7657800
A/duck/Vietnam/LBM/751/2014(H5N6)	EPI596571	EPI596572	EPI596573	EPI596574	EPI596575	EPI596576	EPI596513	EPI596510
A/muscovy duck/Vietnam/LBM/756/2014(H5N6)	EPI596595	EPI596596	EPI596597	EPI596598	EPI596599	EPI596510	EPI596529	EPI596526
A/Guangdong/99710/2014(H5N6)	EPI749879	EPI749878	EPI749877	EPI749881	EPI749874	EPI749880	EPI749876	EPI749875
A/chicken/Shenzhen/2396/2013(H5N6)	KP284986	KP284987	KP284988	KP284989	KP284990	KP284991	KP284992	KP284993
A/Environment/Guangdong/40113/2015(H5N6)	EPI749719	EPI749718	EPI749717	EPI749721	EPI749714	EPI749720	EPI749716	EPI749715
A/environment/Guangdong/GZ670/2015(H5N6)	KU852961	KU852958	KU852955	KU852946	KU852952	KU852949	KU852964	KU852965
A/Yunnan/14564/2015(H5N6)	EPI587627	EPI587626	EPI587625	EPI587629	EPI587622	EPI587628	EPI587624	EPI587623
A/chicken/Laos/LPQ001/2014(H5N6)	KM496962	KM496963	KM496964	KM496965	KM496966	KM496967	KM496968	KM496969
A/duck/Laos/LPQ002/2014(H5N6)				KM496970		KM496971		
A/duck/Guangdong/018/2014(H5N6)	KX094400	KX094401	KX094402	KX094403	KX094404	KX094405	KX094406	KX094407
A/muscovy duck/Vietnam/HU7-23/2017(H5N6)	LC364041	LC364042	LC364043	LC364044	LC364045	LC364046	LC364047	LC364048
A/goose/Yangzhou/YZ587/2016(H5N6)	MF960028	MF960024	MF960020	MF960000	MF960012	MF960008	MF960004	MF960016
A/chicken/Japan/AQ-HE144/2015(H5N6)	LC208494	LC208495	LC208496	LC208492	LC208497	LC208493	LC208498	LC208499
A/duck/Eastern China/S0908/2014(H5N6)				KP732643	KP732663	KP732683	KP732703	KP732723
A/duck/Vietnam/LBM639/2014(H5N1)	AB979508	AB979509	AB979510	AB979511	AB979512	AB979513	AB979514	AB979515
A/duck/Vietnam/LBM638/2014(H5N1)	AB979500	AB979501	AB979502	AB979503	AB979504	AB979505	AB979506	AB979507
A/muscovy duck/Vietnam/LBM631/2014(H5N1)	AB979452	AB979453	AB979454	AB979455	AB979456	AB979457	AB979458	AB979459
A/muscovy duck/Vietnam/LBM635/2014(H5N1)	AB979484	AB979485	AB979486	AB979487	AB979488	AB979489	AB979490	AB979491
A/muscovy duck/Vietnam/LBM636/2014(H5N1)	AB979492	AB979493	AB979494	AB979495	AB979496	AB979497	AB979498	AB979499
A/duck/Jiangsu/m234/2012(H5N2)	JX507352	JX507353	JX507354	JX507355	JX507356	JX507357	JX507358	JX507359

**Strains**	**Genebank code of reference sequences**							
	**PB2**	**PB1**	**PA**	**HA**	**NP**	**NA**	**M**	**NS**
A/Baikal teal/Korea/H52/2014(H5N8)	KJ508963	KJ508964	KJ508965	KJ508961	KJ508966	KJ508962	KJ508967	KJ508968

**Strains**	**Genebank code of reference sequences**							
	**PB2**	**PB1**	**PA**	**HA**	**NP**	**NA**	**M**	**NS**
A/environment/Zhenjiang/C13/2013(H5N6)	KJ938655	KJ938656	KJ938657	KJ938658	KJ938659	KJ938660	KJ938661	KJ938662
A/barn swallow/Hong Kong/1161/2010(H5N1)	KF735641	KF735642	KF735643	KC357320	KF735644	KF735645	KC436130	KF735646
A/duck/Hunan/S4220/2011(H5N1)	CY146697	CY146698	CY146699	CY146700	CY146701	CY146702	CY146703	CY146704
A/chicken/Guangxi/S2039/2009(H5N1)	KT762436	KT762437	KT762438	KT762439	KT762440	KT762441	KT762442	KT762443
A/environment/Guizhou/4/2009(H5N1)	CY098803	CY098804	CY098805	CY098806	CY098807	CY098808	CY098809	CY098810
A/environment/Guizhou/2/2009(H5N1)	CY098795	CY098796	CY098797	CY098798	CY098799	CY098800	CY098801	CY098802
A/avian/Hong Kong/1993/2007(H5N1)	GU050348	GU050347	GU050346	GU050341	GU050344	GU050343	GU050342	GU050345
A/chicken/India/CL03485/2011(H5N1)	CY092139	CY092140	CY092141	CY089470	CY092142	CY089471	CY092143	CY092144
A/chicken/Vietnam/NCVD-KA423/2013(H5N1)	KP097840	KP097861	KP097882	KP097915	KP097938	KP097971	KP097994	KP098015
A/Guangxi/1/2009(H5N1)	CY098741	CY098742	CY098743	CY098744	CY098745	CY098746	CY060162	CY098747
A/Hunan/2/2009(H5N1)	CY098748	CY098749	CY098750	CY098751	CY098752	CY098753	CY060165	CY098754
A/Hunan/1/2009(H5N1)	CY098720	CY098721	CY098722	CY098723	CY098724	CY098725	CY098726	CY060164
A/environment/Guizhou/7/2009(H5N1)	CY098811	CY098812	CY098813	CY098814	CY098815	CY098816	CY098817	CY098818
A/duck/Guangdong/wy19/2008(H5N5)	CY091632	CY091633	CY091634	CY091635	CY091636	CY091637	CY091638	CY091639
A/chicken/Sichuan/81/2005(H5N1)	HM172452	HM172357	HM172309	HM172069	HM172214	HM172167	HM172118	HM172261
A/goose/Guangdong/1/1996(H5N1)		NC_007358	NC_007359	NC_007362			NC_007363	

**Strains**	**Genebank code of reference sequences**							
	**PB2**	**PB1**	**PA**	**HA**	**NP**	**NA**	**M**	**NS**
A/chicken/Shanxi/10/2006 (H5N1)	HM172440	HM172361	HM172337	HM172135	HM172243	HM172172	HM172135	HM172297
A/chicken/Jiangsu/18/2008 (H5N1)	HM172417	HM172368	HM172352	HM172092	HM172234	HM172171	HM172134	HM172275
A/chicken/Hebei/A-8/2009 (H5N1)	HM172411	HM172366	HM172324	HM172081	HM172217	HM172197	HM172137	HM172271
A/chicken/Vietnam/NCVD-swab15/2008(H5N1)				FJ842477				
A/chicken/Vietnam/C58/2004 (H5N1)	AY818127	AY818130	AY818133	AY818136	AY818139	AY818142	AY818145	AY818148
A/chicken/Vietnam/NCVD-swab17/2008(H5N1)				FJ842478				
A/ck/Indonesia/PA/2003 (H5N1)	AY651705	AY651653	AY651602	AY651320	AY651487	AY651433	AY651376	AY651541
A/Indonesia/CDC940/2006 (H5N1)	CY017651	CY017652	CY017653	CY017654	CY017655	CY017656	CY017657	CY017658
A/Indonesia/CDC887/2006 (H5N1)	CY017685	CY017686	CY017687	CY017688	CY017689	CY017690	CY017691	CY017692
A/Indonesia/CDC595/2006 (H5N1)	CY014277	CY014278	CY014279	CY014280	CY014281	CY014282	CY014283	CY014284
A/Indonesia/546H/2006 (H5N1)	EU146776	EU146773	EU146770	EU146755	EU146764	EU146761	EU146758	EU146767
A/bar/headed/goose/Qinghai/5/2005 (H5N1)	DQ095757	DQ095737	DQ095717	DQ095617	DQ095677	DQ095657	DQ095637	DQ095697

**Strains**	**Genebank code of reference sequences**							
	**PB2**	**PB1**	**PA**	**HA**	**NP**	**NA**	**M**	**NS**
A/turkey/Turkey/1/2005 (H5N1)	EF619975	EF619976	EF619979	EF619980	EF619977	EF619973	EF619978	EF619974
A/tundra swan/Mongolia/1T/2010(H5N1)				CY098292				
A/Vietnam/1194/2004 (H5N1)				GQ149237				
A/duck/Hunan/1265/2005 (H5N1)	DQ320845	DQ321306	DQ321240	DQ320911	DQ321108	DQ321043	DQ320977	DQ321174
**Strains**	**Genebank code of reference sequences**							
	**PB2**	**PB1**	**PA**	**HA**	**NP**	**NA**	**M**	**NS**
A/goose/Guangxi/345/2005 (H5N1)	DQ320830	DQ321291	DQ321225	DQ320896	DQ321093	DQ321028	DQ320962	DQ321159
A/duck/Hunan/149/2005 (H5N1)	DQ320838	DQ321299	DQ321233	DQ320904	DQ321101	DQ321036	DQ320970	DQ321167
A/duck/Yunnan/4400/2005 (H5N1)	DQ992665	EF123991	EF124746	DQ992797	EF124444	EF124293	EF124142	EF124595
A/duck/Hunan/139/2005 (H5N1)	DQ320837	DQ321298	DQ321232	DQ321035	DQ321100	DQ321035	DQ320969	DQ321166
A/chicken/Hunan/999/2005 (H5N1)	DQ320844	DQ321305	DQ321239	DQ320910	DQ321107	DQ321042	DQ320976	DQ321173
A/goose/Guiyang/3422/2005 (H5N1)	DQ992679	EF124005	EF124760	DQ992757	EF124458	EF124307	EF124156	EF124609
A/chicken/Guiyang/3055/2005 (H5N1)	DQ992677	EF124003	EF124758	DQ992755	EF124456	EF124305	EF124154	EF124607
A/duck/Guiyang/3242/2005 (H5N1)	DQ992678	EF124004	EF124759	DQ992756	EF124457	EF124306	EF124155	EF124608
A/goose/Yunnan/6193/2006 (H5N1)	CY030974	CY030975	CY030976	CY030977	CY030978	CY030979	CY030980	CY030981
A/Muscovy/duck/Vietnam/39/2007(H5N1)	CY029668	CY029669	CY029670	CY029671	CY029672	CY029673	CY029674	CY029675
A/Muscovy duck/Vietnam/41/2007 (H5N1)	CY029676	CY029677	CY029678	CY029679	CY029680	CY029681	CY029682	CY029683
A/chicken/Vietnam/NCVD-20/2007 (H5N1)	CY030380	CY030381	CY030382	CY030383	CY030384	CY030385	CY030386	CY030387
A/avian/Hong Kong/2372/2007 (H5N1)	GU050340	GU050339	GU050338	GU050333	GU050336	GU050335	GU050334	GU050337
A/Anhui/1/2005 (H5N1)	HM172438	HM172394	HM172342	HM172104	HM172254	HM172189	HM172159	HM172266
A/common magpie/Hong Kong/645/2006 (H5N1)	DQ992575	EF123901	EF124656	DQ992839	EF124354	EF124203	EF124052	EF124505
A/duck/Vietnam/NCVD/1584/2012(H5N1)	EPI424981	EPI424982	EPI424980	EPI424984	EPI424977	EPI424983	EPI424979	EPI424978
**Strains**	**Genebank code of reference sequences**							
	**PB2**	**PB1**	**PA**	**HA**	**NP**	**NA**	**M**	**NS**
A/bar-headed goose/Mongolia/X53/2009 (H5N1)	KR732547	KR732560	KR732516	KR732516	KR732495	KR732495	KR732563	KR732555
A/tundra swan/Mongolia/1T/2010				CY098292				
A/chicken/Bangladesh/15078/2012(H5N1)	KF888543	KF888544	KF888545	KF888546	KF888547	KF888548	KF888549	KF888550
A/duck/Bangladesh/19097/2013(H5N1)	KF874284	KF874285	KF874286	KF715205	KF874287	KF715206	KF874288	KF874289
A/duck/Vietnam/NCVD/129/7/2011/(H5N1)	EPI425197	EPI425198	EPI425196	EPI425200	EPI425193	EPI425199	EPI425195	EPI425194
A/duck/Vietnam/NCVD/672/2011(H5N1)				EPI620817	EPI620838		EPI622554	EPI620973
A/duck/Vietnam/NCVD/920/2011/H5N1	EPI424885	EPI424886	EPI424884	EPI424888	EPI424881	EPI424887	EPI424883	EPI424882
A/duck/Vietnam/NCVD/927/2011/H5N1	EPI425485	EPI425486	EPI438916	EPI425488	EPI425481	EPI425487	EPI425483	EPI425482
A/turkey/Turkey/1/2005(H5N1)				KF042153				

**Strains**	**Genebank code of reference sequences**							
	**PB2**	**PB1**	**PA**	**HA**	**NP**	**NA**	**M**	**NS**
A/bar/headed/goose/Qinghai/5/2005(H5N1)				DQ095617				
A/chicken/Hebei/A-8/2009(H5N1)				HM172081				
A/chicken/Jiangsu/18/2008(H5N1)				HM172092				
A/chicken/Shanxi/10/2006(H5N1)				HM172113				
A/teal/Tottori/1/2016(H5N6)	LC199865	LC199866	LC199867	LC199868	LC199869	LC199870	LC199871	LC199872
A/duck/Vietnam/LBM632/2014(H5N1)	AB979460	AB979461	AB979462	AB979463	AB979464	AB979465	AB979466	AB979467
A/duck/Vietnam/LBM633/2014(H5N1)	AB979468	AB979469	AB979470	AB979471	AB979472	AB979473	AB979474	AB979475
**Strains**	**Genebank code of reference sequences**							
	**PB2**	**PB1**	**PA**	**HA**	**NP**	**NA**	**M**	**NS**
A/duck/Jiangxi/JXA131996/2013(H5N2)	KM234807		KM234808		KM234809		KM234810	KM234811
A/duck/Laos/XBY004/2014(H5N6)	KM496974	KM496975	KM496976	KM496977	KM496978	KM496979	KM496980	KM496981
A/goose/Vietnam/NCVD-15A27/2015(H5N6)	KY171691							
A/chicken/Vietnam/NCVD-15A55/2015(H5N6)	KY171706							
A/duck/Vietnam/LBM568/2014(H5N1)	AB972688	AB972689	AB972690	AB972691	AB972692	AB972693	AB972694	AB972695
A/muscovy duck/Vietnam/LBM567/2014(H5N1)	AB972680	AB972681	AB972682	AB972683	AB972684	AB972685	AB972686	AB972687
A/bar-headed goose/Qinghai/5/2005(H5N1)	DQ095757	DQ095737	DQ095717	DQ095617	DQ095677	DQ095657	DQ095637	DQ095697
A/turkey/Turkey/1/2005(H5N1)	EF619975	EF619976	EF619979		EF619977		EF619978	EF619974
A/duck/Fujian/11094/2005(H5N1)	DQ992636	EF123962	EF124717		EF124415		EF124113	EF124566
A/Vietnam/1194/2004(H5N1)				GQ149237				
A/Indonesia/CDC887/2006(H5N1)	CY017685	CY017686	CY017687		CY017689		CY017691	CY017692
A/Indonesia/CDC940/2006(H5N1)	CY017651	CY017652	CY017653		CY017655		CY017657	CY017658
A/Indonesia/546H/2006(H5N1)	EU146776	EU146773	EU146770		EU146764		EU146758	EU146767
A/Indonesia/CDC595/2006(H5N1)	CY014277	CY014278	CY014279		CY014281		CY014283	CY014284
A/chicken/Indonesia/PA/2003(H5N1)			AY651602					
A/duck/Eastern China/108/2008(H5N1)	GU727666	GU727667	GU727668					
**Strains**	**Genebank code of reference sequences**							
	**PB2**	**PB1**	**PA**	**HA**	**NP**	**NA**	**M**	**NS**
A/duck/Eastern China/031/2009(H5N5)	GU727658	GU727659	GU727660					
A/duck/Guangdong/wy24/2008(H5N5)	CY091640	CY091641	CY091642					
A/duck/Guangdong/wy11/2008(H5N5)	CY091624	CY091625	CY091626					
A/peregrine falcon/Hokkaido/X7/2016(H5N6)	LC317075	LC317076	LC317077	LC317078	LC317079	LC317080	LC317081	LC317082
A/whooper swan/Niigata/13/2017(H5N6)	LC318925	LC318926	LC318927	LC318928	LC318929	LC318930	LC318931	LC318932
A/duck/Vietnam/LBM751/2014	LC028189	LC028190	LC028191	LC028192	LC028193	LC028194	LC028195	LC028196

**Strains**	**Genebank code of reference sequences**							
	**PB2**	**PB1**	**PA**	**HA**	**NP**	**NA**	**M**	**NS**
A/muscovy duck/Vietnam/LBM755/2014(H5N6)	LC028304	LC028305	LC028306	LC028307	LC028308	LC028309	LC028310	LC028311
A/muscovy duck/Vietnam/LBM754/2014(H5N6)	LC028205	LC028206	LC028207	LC028208	LC028209	LC028210	LC028211	LC028212
A/muscovy duck/Vietnam/LBM756/2014(H5N6)	LC028312	LC028313	LC028314	LC028315	LC028316	LC028317	LC028318	LC028319
A/duck/Vietnam/LBM752/2014(H5N6)	LC028197	LC028198	LC028199	LC028200	LC028201.	LC028202	LC028203	LC028204
A/duck/jiangxi/JXA132023/2013(H5N2)	KM234812	KM234813	KM234814		KM234815		KM234816	KM234817
A/duck/Vietnam/LBM360c1-4-1/2013(H5N6)	LC010693	LC010694	LC010695	LC010696	LC010697	LC010698	LC010699	LC010700
A/chicken/Vieynam/C58/2004(H5N1)		AY818130						
A/environment/Guangdong/GZ693/2015(H5N6)	KU852959	KU852956	KU852953	KU852944	KU852950	KU852947	KU852962	KU852966
A/chicken/Heilongjiang/S7/2014(H5N2)	KX160178	KX160177	KX160176	KX160179	KX160174	KX160173	KX160172	KX160175
A/chicken/Shenzhen/2464/2013(H5N6)	KP284994	KP284995	KP284996	KP284997	KP284998	KP284999	KP285000	KP285001
**Strains**	**Genebank code of reference sequences**							
	**PB2**	**PB1**	**PA**	**HA**	**NP**	**NA**	**M**	**NS**
A/duck/Vietnam/LBM751/2014(H5N6)	LC028189	LC028190	LC028191	LC028192	LC028193	LC028194	LC028195	LC028196
A/duck/Vietnam/NCVD-A672/2011(H5N1)			KP097888					
A/Northern/Pintail/Tottori/b37/2016(H5N6)	LC200414	LC200415	LC200416	LC200417	LC200418	LC200419	LC200420	LC200421
A/whooper swan/Korea/Gangjin/48/2016(H5N6)	KY402046	KY402047	KY402048	KY402049	KY402050	KY402051	KY402046	KY402052
A/teal/Tottori/2/2016(H5N6)	LC200933	LC200934	LC200935	LC200936	LC200937	LC200938	LC200939	LC200940
A/chicken/Dongguan/3363/2013(H5N6)	KP286106	KP286107	KP286108	KP286109	KP286110	KP286111	KP286112	KP286113
A/muscovy duck/Vietnam/LBM634/2014(H5N1)	AB979476	AB979477	AB979478	AB979479	AB979480	AB979481	AB979482	AB979483
A/China/GD01/2006(H5N1)	DQ835310	DQ835311	DQ835312	DQ835313	DQ835314	DQ835315	DQ835316	DQ835317
A/duck/Guangzhou/021/2014(H5N6)	KX094415	KX094414	KX094413	KX094412	KX094411	KX094410	KX094409	KX094408
A/environment/Guangdong/JY137/2014(H5N6)	KT370111	KT370105	KT370098	KT370064	KT370084	KT370074	KT370071	KT370092
A/chicken/Shenzhen/1061/2013(H5N6)	KP286082	KP286083	KP286084	KP286085	KP286086	KP286087	KP286088	KP286089
A/duck/Guangzhou/018/2014(H5N6)	KX094407	KX094406	KX094405	KX094400	KX094403	KX094402	KX094401	KX094404
A/goose/Hunan/118/2014(H5N6)	KX121195	KX121196	KX121197	KX121198	KX121199	KX121200	KX121201	KX121202
A/Shenzhen/1/2011(H5N1)							KC436112	
A/brown headed gull/Hong Kong/709/2011(H5N1)							KC436117	
A/duck/Zhejiang/213/2011(H5N1)							JN646734	
**Strains**	**Genebank code of reference sequences**							
	**PB2**	**PB1**	**PA**	**HA**	**NP**	**NA**	**M**	**NS**
A/duck/Zhejiang/224/2011(H5N1)							JN646735	
A/large billed crow/Hong Kong/497/2011(H5N1)							KC436118	
A/duck/Vietnam/LBM760/2014(H5N6)	LC028344	LC028345	LC028346	LC028347	LC028348	LC028349	LC028350	LC028351

**Strains**	**Genebank code of reference sequences**							
	**PB2**	**PB1**	**PA**	**HA**	**NP**	**NA**	**M**	**NS**
A/muscovy/duck/Vietnam/LBM757/2014(H5N6)	LC028320	LC028321	LC028322	LC028323	LC028324	LC028325	LC028326	LC028327
A/chicken/Jiangxi/NCDZT1123/2014(H5N6)	KP090436	KP090437	KP090438	KP090439	KP090440	KP090441	KP090442	KP090443
A/duck/Hubei/WH18/2015(H5N6)				KX652135		KX652136		
A/duck/Hubei/XG18/2015(H5N6)				KX652134		KX652138		
A/chicken/Hubei/XG18/2015(H5N6)				KX652133		KX652137		
A/duck/Dongguan/2685/2013	KP285002	KP285003	KP285004	KP285005	KP285006	KP285007	KP285008	KP285009
A/duck/Guangxi/175D12/2014(H3N6)	KR919745	KR919744	KR919743	KR919740	KR919742	KR919741	KR919747	KR919746
A/Gallinula/chloropus/Guangdong/GZ174(H5N6)	KT454944	KT454945	KT454946	KT454947	KT454948	KT454950	KT454951	KT454952
A/chicken/Jiangxi/14512/2014(H10N6)	KP286442	KP286443	KP286444	KP286445	KP286446	KP286447	KP286448	KP286449
A/chicken/Jiangxi/13202/2014(H10N6)	KP285954	KP285955	KP285956	KP285957	KP285958	KP285959	KP285960	KP285961
A/chicken/Jiangxi/12782/2014(H10N6)	KT370051	KT370047	KT370034	KT369983	KT370014	KT370001	KT369993	KT370021
A/duck/Guangdong/S4251/2010(H6N6)	KJ200828	KJ200827	KJ200826	KJ200821	KJ200824	KJ200823	KJ200822	KJ200825
A/swine/Guangdong/K6/2010(H6N6)	HM804480	HM804479	HM804478	HM800947	HM804477	HM804474	HM804476	HM804475

**Strains**	**Genebank code of reference sequences**							
	**PB2**	**PB1**	**PA**	**HA**	**NP**	**NA**	**M**	**NS**
A/duck/Guangdong/S1419/2011(H6N6)	KJ200748	KJ200747	KJ200746	KJ200745	KJ200744	KJ200743	KJ200742	KJ200741
A/duck/Shantou/1984/2007(H6N6)	CY109759	CY109760	CY109761	CY109762	CY109763	CY109764	CY109765	CY109766
A/mallard/Shantou/972/2005(H6N6)	HM145728	HM145559	HM145390	HM144544	HM145221	HM144714	HM144883	HM145052
A/duck/Shantou/9350/2006(H6N6)				CY110313		CY110314		
A/duck/Fujian/958/2006(H6N6)	CY109487	CY109488	CY109489	CY109490	CY109491	CY109492	CY109493	CY109494
A/duck/Fujian/3242/2007(H6N6)				CY110125		CY110126		
A/goose/Guangdong/S4362/2009(H6N6)				KJ200933		KJ200935		
A/duck/Guangdong/S3468/2010(H6N6)	KJ200804	KJ200803	KJ200802	KJ200797	KJ200800	KJ200799	KJ200798	KJ200801
A/chicken/Guangdong/S1311/2010(H6N6)				KJ200637		KJ200639		
A/chicken/Guangdong/S1414/2010(H6N6)	KJ200660	KJ200659	KJ200658	KJ200653	KJ200656	KJ200655	KJ200654	KJ200657
A/duck/Hunan/S4273/2010(H6N6)						KJ200887		
A/duck/Guangdong/S1155/2011(H6N6)						KJ200767		
A/duck/Guangdong/S3073/2010(H6N6)						KJ200775		
A/duck/Zhejiang/S4204/2010(H6N6)						KJ200919		
A/duck/Guangdong/S3225/2010(H6N6)						KJ200791		
A/duck/Guangdong/S4018/2010(H6N6)						KJ200807		

**Strains**	**Genebank code of reference sequences**							
	**PB2**	**PB1**	**PA**	**HA**	**NP**	**NA**	**M**	**NS**

**Strains**	**Genebank code of reference sequences**							
	**PB2**	**PB1**	**PA**	**HA**	**NP**	**NA**	**M**	**NS**
A/duck/Zhejiang/S1134/2011(H6N6)						KJ200911		
A/duck/Potsdam/2216-4/1984(H5N6)						CY005771		
A/turkey/Minnesota/957/80(H6N6)						AY207547		
A/mallard/California/1500P/2013(H5N6)						CY177427		
A/mallard/California/940V/2013(H4N6)						CY177103		
A/mallard/California/937V/2013(H4N6)						CY177095		
A/mallard/California/1418/2013(H5N6)						CY176975		
A/northern/pintail/Alberta/265/2007(H4N6)						CY103342		
A/northern shoveler/Oregon/44336-179/2007(H4N6)						CY076471		
A/blue-winged teal/Louisiana/Sg-00189/2007(H3N6)						CY078148		
A/blue-winged teal/Texas/Sg-00085/2007(H3N6)						CY078259		
A/wild duck/Shandong/628/2011(H5N1)	EPI475576	EPI475577	EPI475578	EPI475579	EPI475580	EPI475581	EPI475582	EPI475583
A/duck/Eastern China/1111/2011(H5N2)	EPI398953	EPI398957	EPI398961	EPI398965	EPI398969	EPI398973	EPI398977	EPI403396
A/chicken/Ghana/46/2015 (H5N1)	EPI770686	EPI770687	EPI770685	EPI770680	EPI770682	EPI770681	EPI770684	EPI770683
